# Supplementary figures and images for: Whole-transcriptome analysis reveals mechanisms underlying antibacterial activity and biofilm inhibition by a malic acid combination (MAC) in Pseudomonas aeruginosa
Source: PeerJ. 2023 Dec 7;11:e16476. doi: 10.7717/peerj.16476 (PMC10710775; doi:10.7717/peerj.16476)

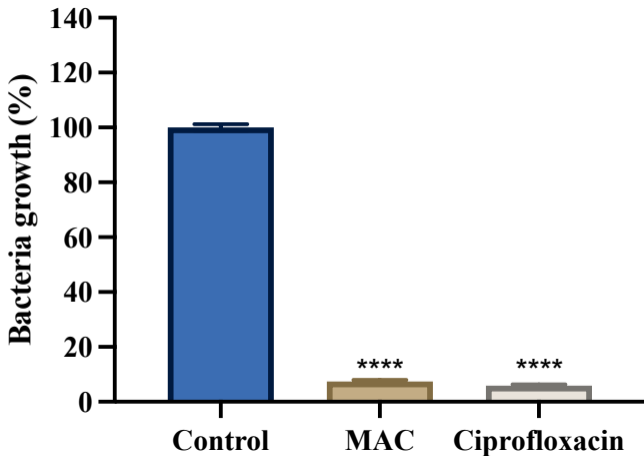

Supplement: Supplemental Information 2 [file peerj-11-16476-s002.pdf]

**A**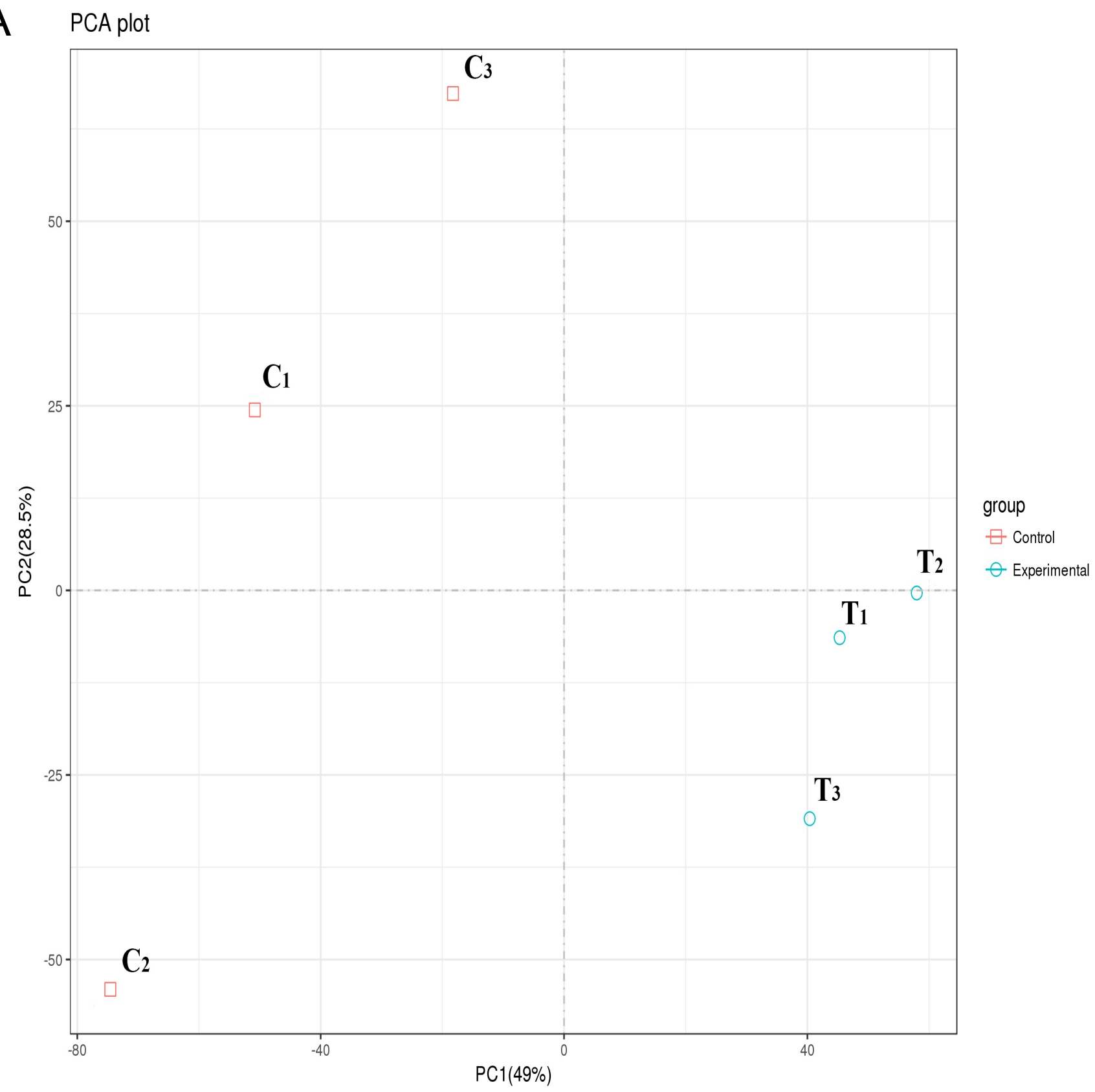**B**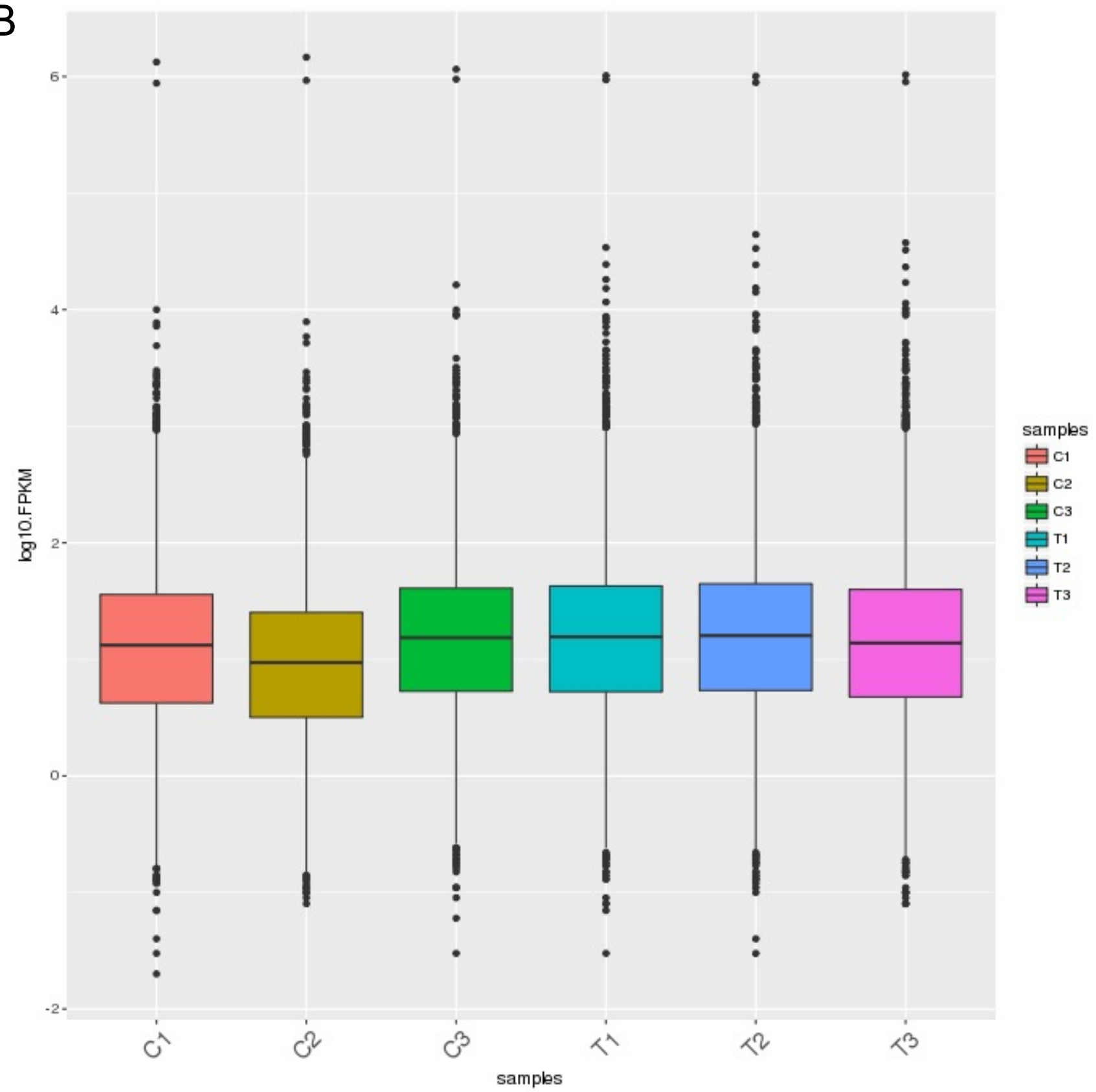

Supplement: Supplemental Information 3 — (A) Correlation analysis of samples in the control group and the treated group. Bi-plot of the principal component analysis of DESeq2 normalized read counts (all coding genes) for treatment (green) and the control (red), split into technical replicates. (B) FPKM Boxplot Analysis. Each boxplot region depicts five statistical measures, including the maximum value, upper quartile (Q3), median (Q2), lower quartile (Q1), and minimum value. [file peerj-11-16476-s003.pdf]

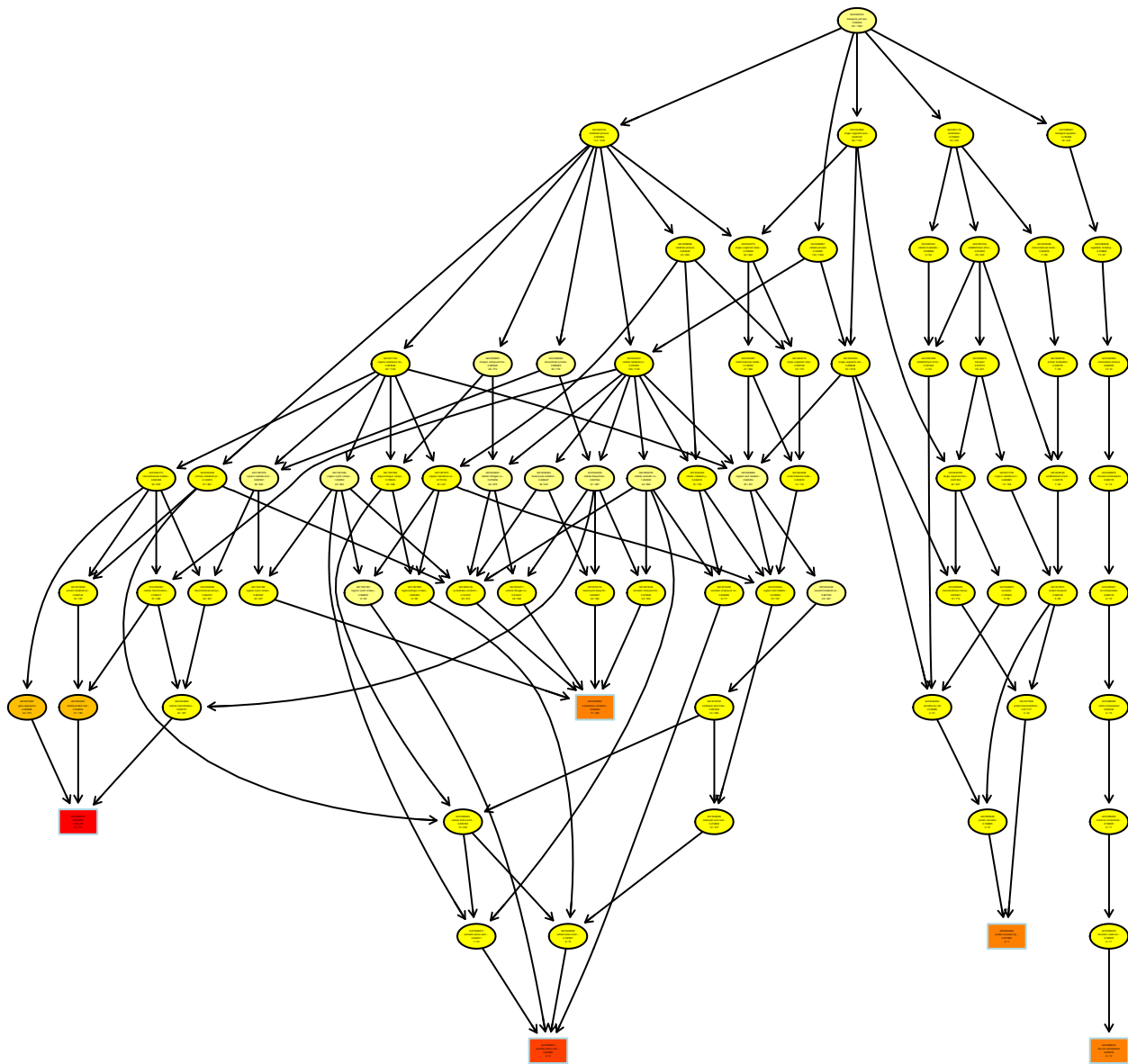

Supplement: Supplemental Information 4 — The DAG of BP in the up-regulated gene GO term [file peerj-11-16476-s004.pdf]

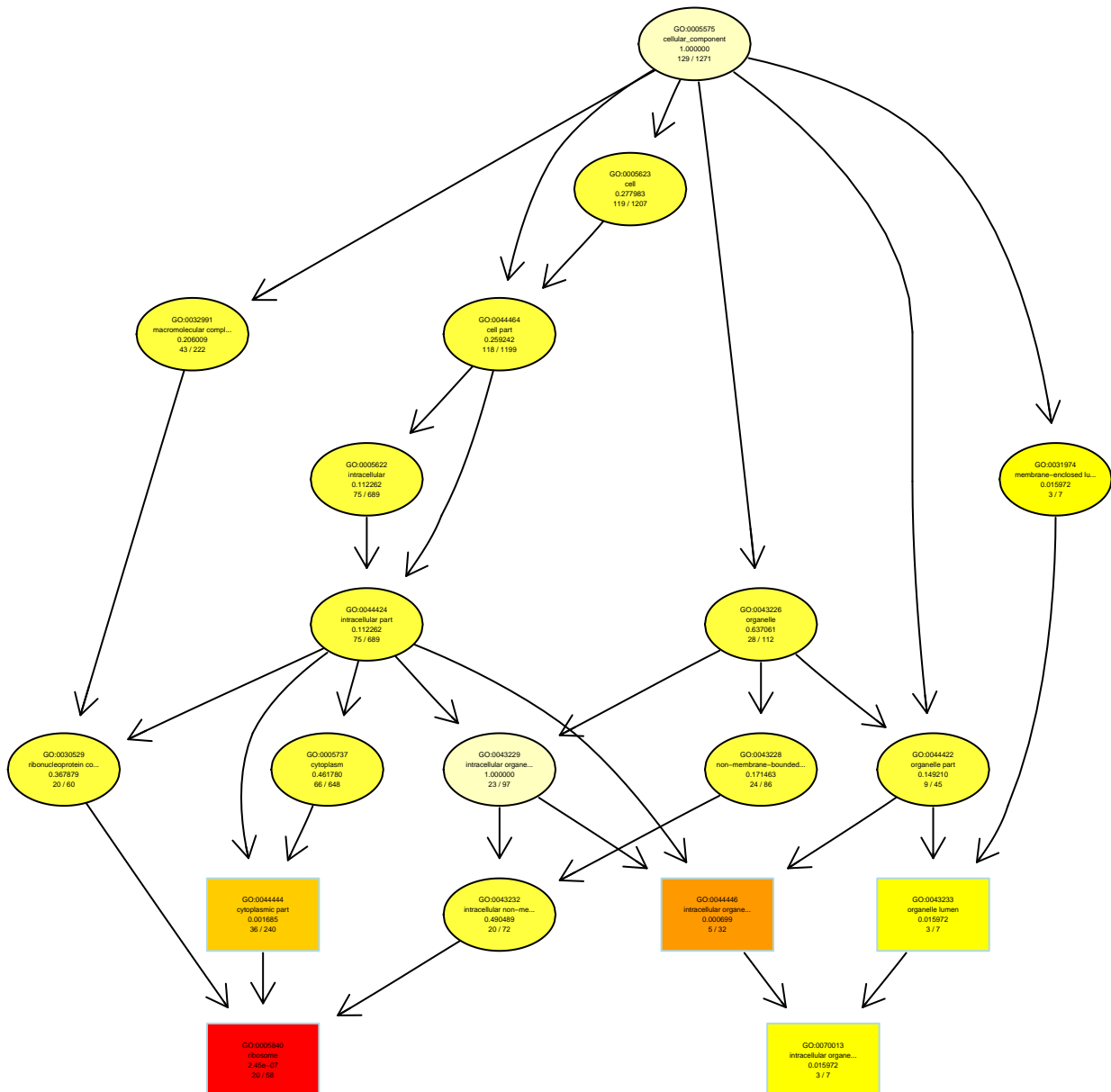

Supplement: Supplemental Information 5 — The DAG of CC in the up-regulated gene GO term. [file peerj-11-16476-s005.pdf]

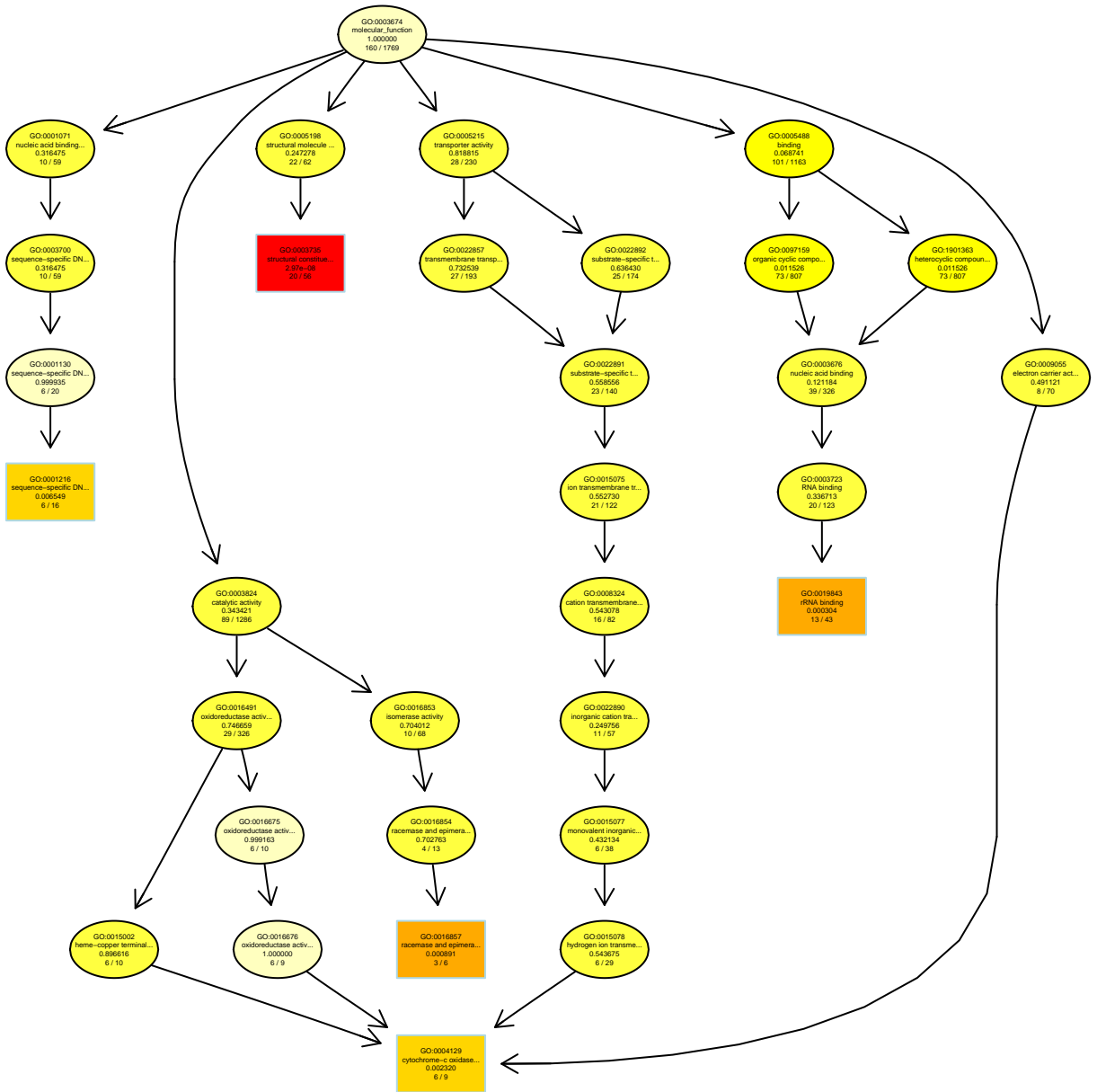

Supplement: Supplemental Information 6 — The DAG of MF in the up-regulated gene GO term. [file peerj-11-16476-s006.pdf]

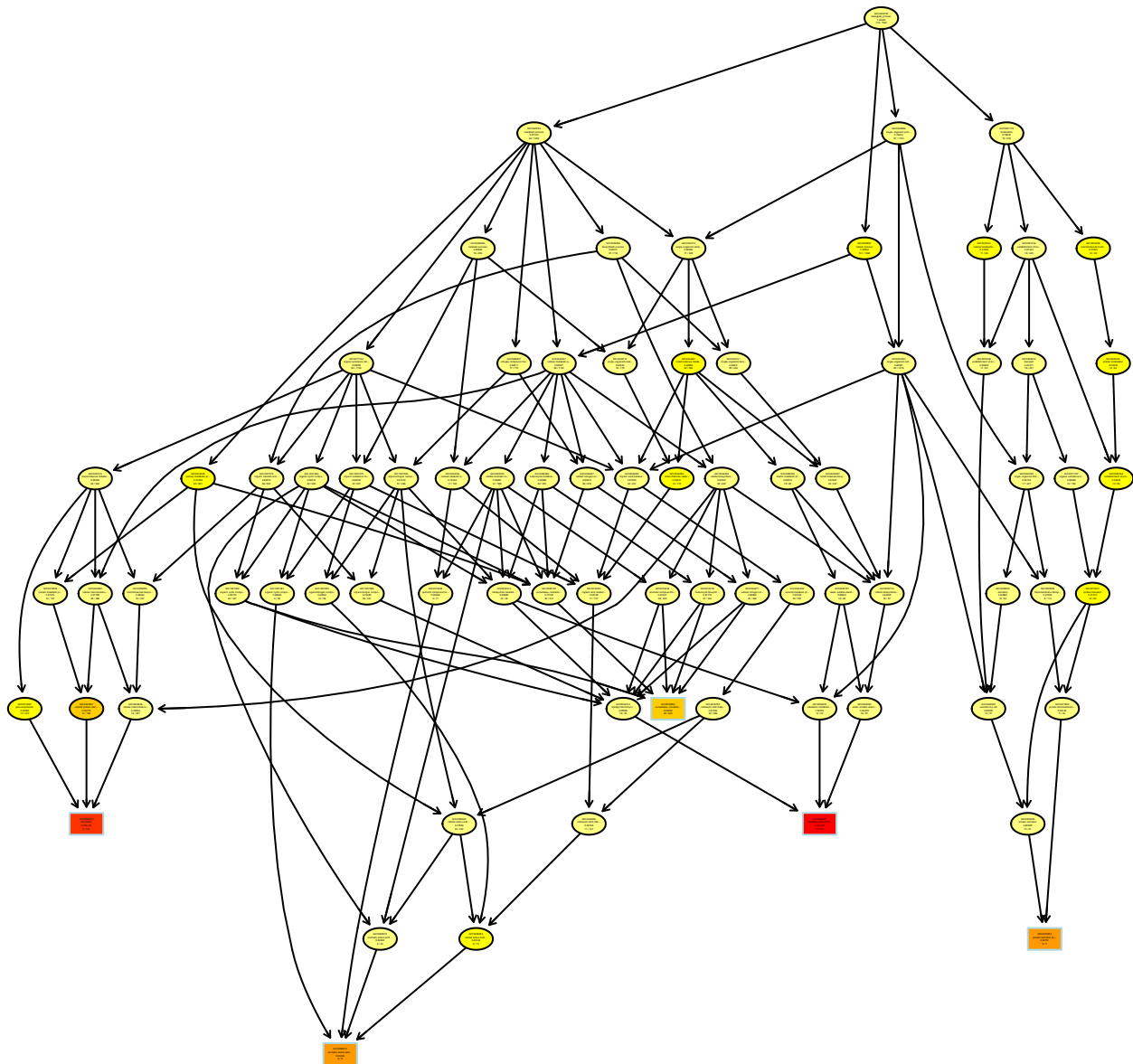

Supplement: Supplemental Information 7 — The DAG of BP in the down-regulated gene GO term [file peerj-11-16476-s007.pdf]

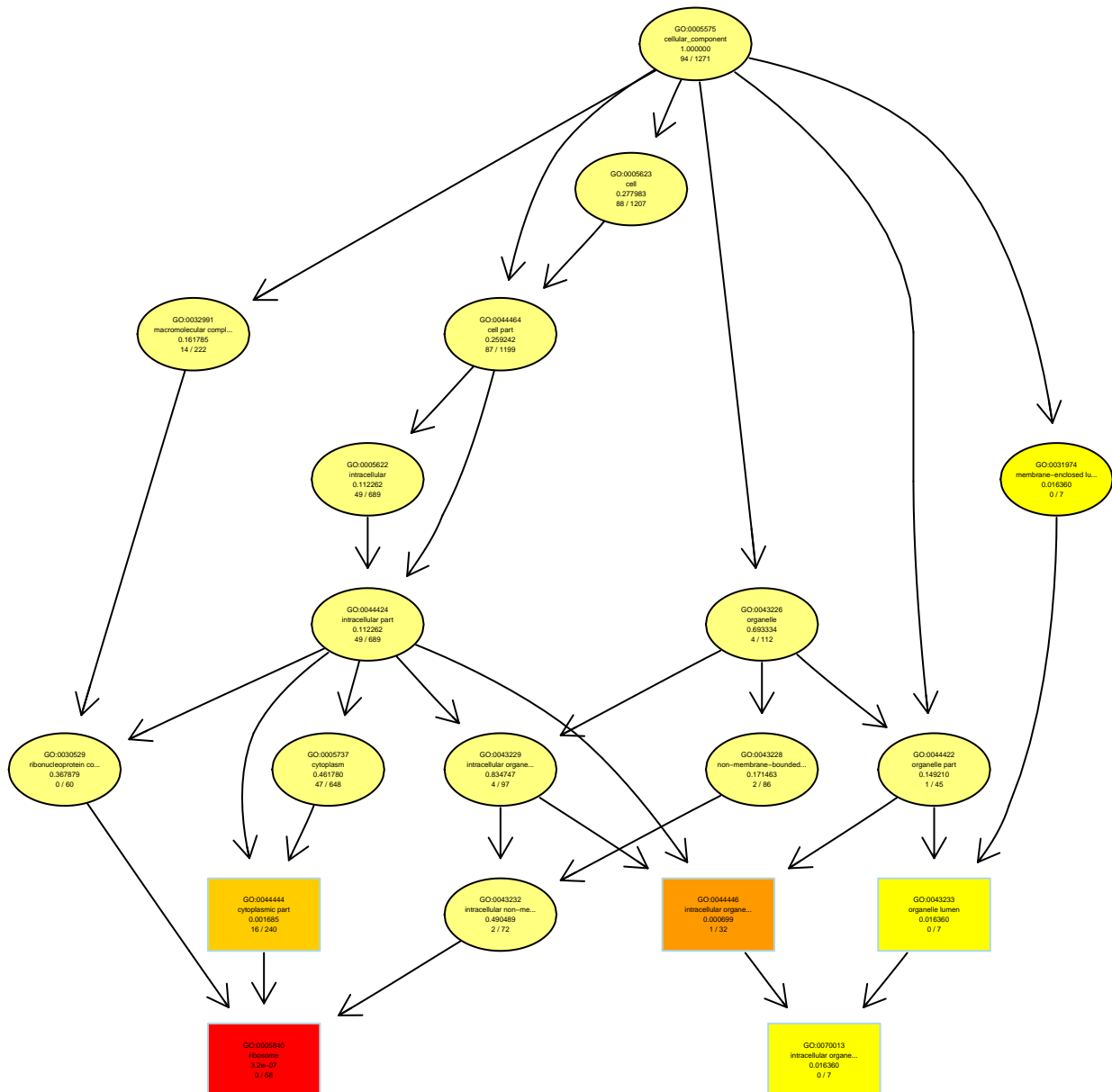

Supplement: Supplemental Information 8 — The DAG of CC in the down-regulated gene GO term [file peerj-11-16476-s008.pdf]

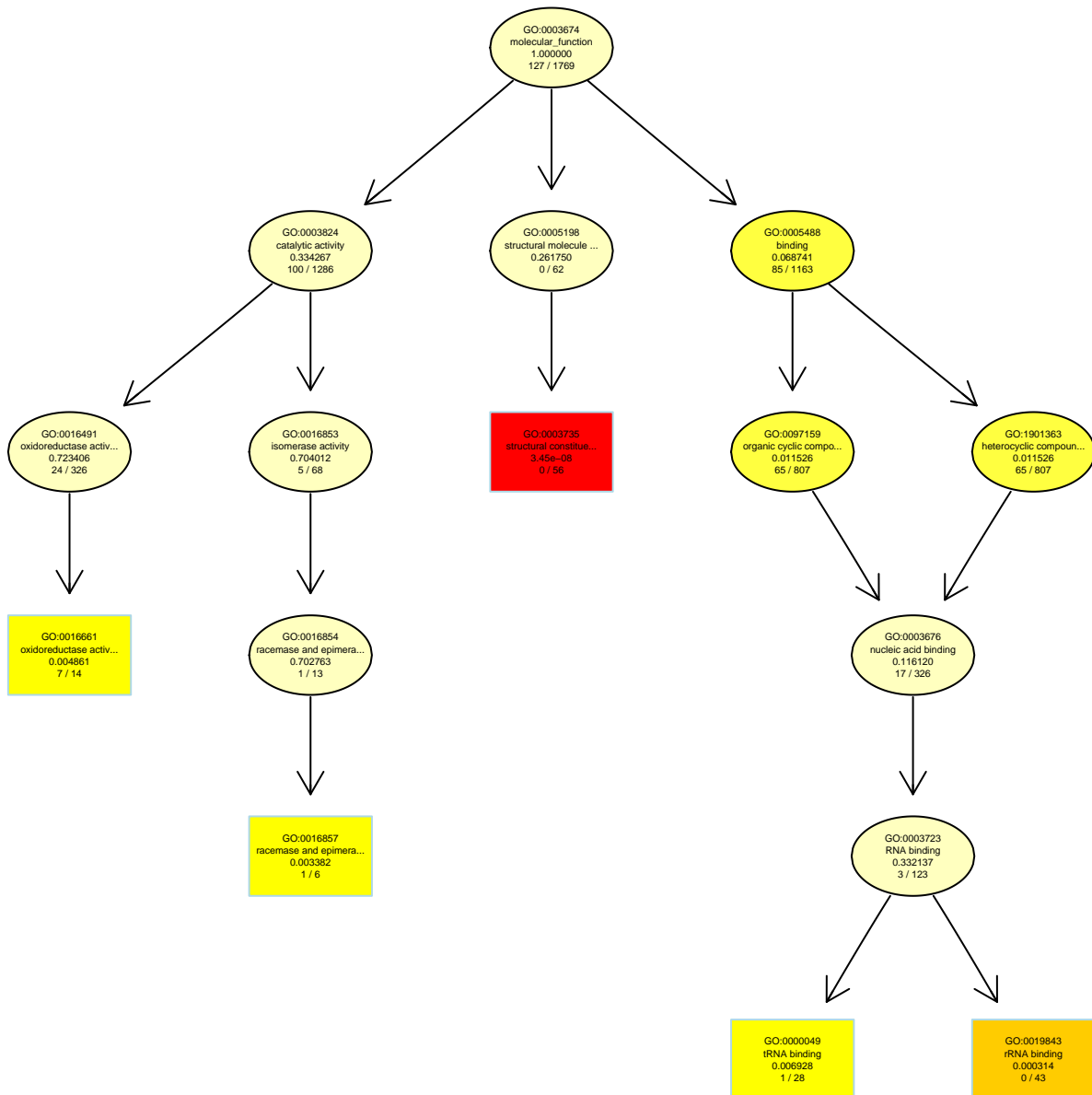

Supplement: Supplemental Information 9 — The DAG of MF in the down-regulated gene GO term [file peerj-11-16476-s009.pdf]

BACTERIAL SECRETION SYSTEM

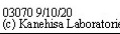

PORPHYRIN METABOLISM

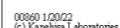

TWO-COMPONENT SYSTEM

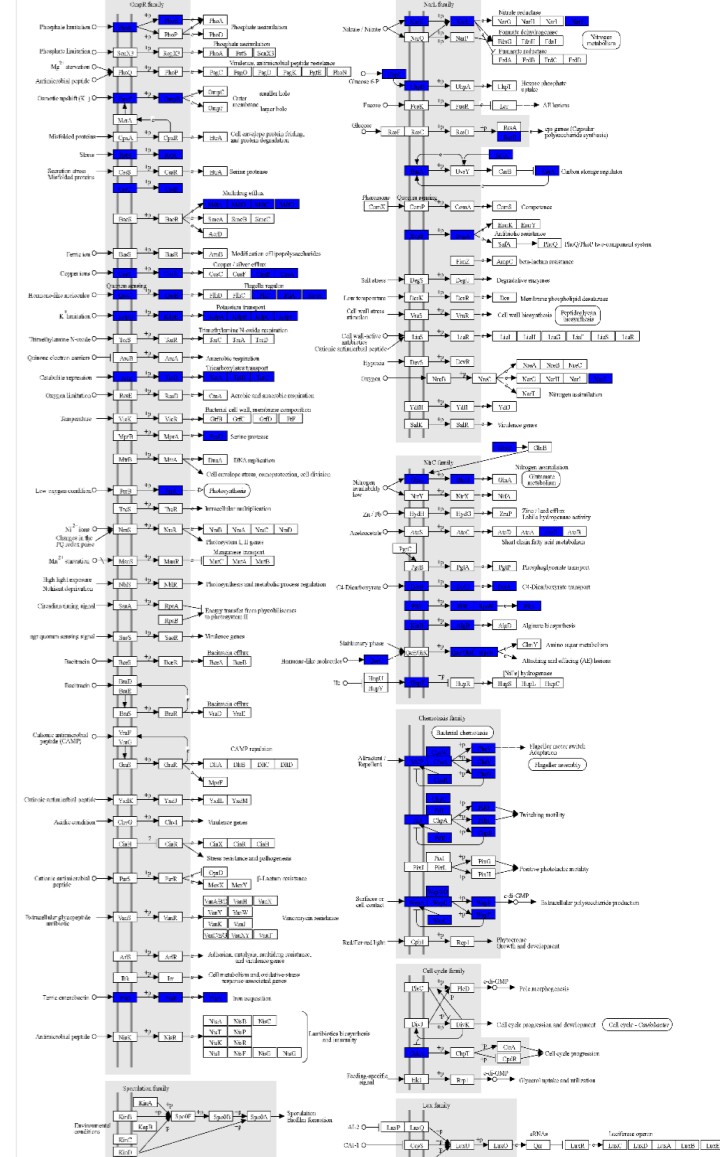

Supplement: Supplemental Information 11 — (A) Bacterial secretion system, (B) Porphyrin metabolism, (C) Two-component system. The blue box indicate that the RNA expression of the gene is down-regulated, while the black box shows no changes in gene RNA expression [file peerj-11-16476-s011.pdf]
