# Supplementary material for: Whole-transcriptome analysis reveals mechanisms underlying antibacterial activity and biofilm inhibition by a malic acid combination (MAC) in Pseudomonas aeruginosa
Source: PeerJ. 2023 Dec 7;11:e16476. doi: 10.7717/peerj.16476 (PMC10710775; doi:10.7717/peerj.16476)
Supplement: Supplemental Information 10 — (A) Ribosome, (B) aminoacyl-tRNA biosynthesis. The red box indicate that the RNA expression of the gene is down-regulated, while the black box shows no changes in gene RNA expression [file peerj-11-16476-s010.pdf]

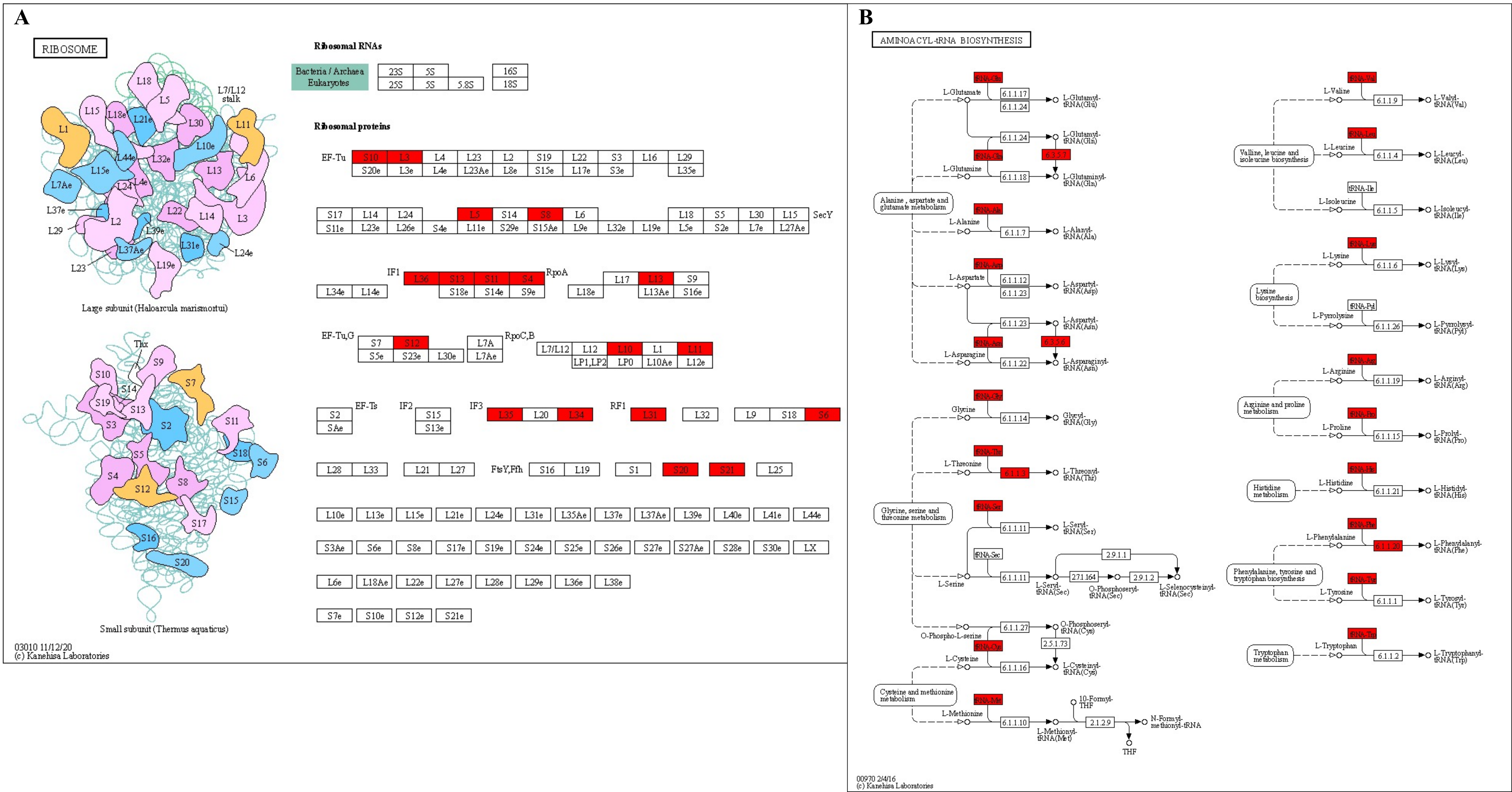
A
RIBOSOME
Ribosomal RNAs
Bacteria / Archaea
Eukaryotes
23S 5S 16S
25S 5S 5.8S 18S
Ribosomal proteins
EF-Tu
S10 L3 L4 L23 L2 S19 L22 S3 L16 L29
S20e L3e L4e L23Ae L8e S15e L17e S3e L35e
S17 L14 L24 L5 S14 S8 L6 L18 S5 L30 L15 SecY
S11e L23e L26e S4e L11e S29e S15Ae L9e L32e L19e L5e S2e L7e L27Ae
IF1 L36 S13 S11 S4 RpoA
L34e L14e S18e S14e S9e
L17 L13 S9
L18e L13Ae S16e
EF-Tu,G S7 S12 L7A RpoC,B
S5e S23e L30e L7Ae
L7/L12 L12 L10 L1 L11
LP1,LP2 LP0 L10Ae L12e
S2 EF-Ts IF2 S15 IF3 L35 L20 L34 RF1 L31 L32 L9 S18 S6
SAe S13e
L28 L33 L21 L27 FtsY,Ffh S16 L19 S1 S20 S21 L25
L10e L13e L15e L21e L24e L31e L35Ae L37e L37Ae L39e L40e L41e L44e
S3Ae S6e S8e S17e S19e S24e S25e S26e S27e S27Ae S28e S30e LX
L6e L18Ae L22e L27e L28e L29e L36e L38e
S7e S10e S12e S21e
L7/L12 stalk
L18 L5 L15 L18e L21e L30 L1 L11 L10e L44e L32e L15e L13 L6 L7Ae L24 L4e L37e L22 L14 L3 L2 L29 L39e L31e L37Ae L24e L23 L19e
Large subunit (Haloarcula marismortui)
Thx S9 S10 S14 S7 S19 S13 S2 S11 S3 S5 S18 S6 S4 S8 S12 S15 S17 S16 S20
Small subunit (Thermus aquaticus)
03010 11/12/20
(c) Kanehisa Laboratories
B
AMINOACYL-tRNA BIOSYNTHESIS
L-Glutamate 6.1.1.17 6.1.1.24 L-Glutamyl-tRNA(Glu)
6.1.1.24 L-Glutamyl-tRNA(Gln) 6.3.5.7
L-Glutamine 6.1.1.18 L-Glutaminyl-tRNA(Gln)
Alanine, aspartate and glutamate metabolism
L-Alanine 6.1.1.7 L-Alanyl-tRNA(Ala)
L-Aspartate 6.1.1.12 6.1.1.23 L-Aspartyl-tRNA(Asp)
6.1.1.23 L-Aspartyl-tRNA(Asn) 6.3.5.6
L-Asparagine 6.1.1.22 L-Asparaginyl-tRNA(Asn)
Glycine 6.1.1.14 Glycyl-tRNA(Gly)
L-Threonine 6.1.1.3 L-Threonyl-tRNA(Thr)
Glycine, serine and threonine metabolism
6.1.1.11 L-Seryl-tRNA(Ser)
tRNA-Sec
2.9.1.1
L-Serine 6.1.1.11 L-Seryl-tRNA(Sec) 2.7.1.164 O-Phosphoseryl-tRNA(Sec) 2.9.1.2 L-Selenocysteinyl-tRNA(Sec)
O-Phospho-L-serine 6.1.1.27 O-Phosphoseryl-tRNA(Cys) 2.5.1.73
L-Cysteine 6.1.1.16 L-Cysteinyl-tRNA(Cys)
Cysteine and methionine metabolism
L-Methionine 6.1.1.10 L-Methionyl-tRNA(Met) 10-Formyl-THF 2.1.2.9 N-Formyl-methionyl-tRNA THF
L-Valine 6.1.1.9 L-Valyl-tRNA(Val)
Valine, leucine and isoleucine biosynthesis
L-Leucine 6.1.1.4 L-Leucyl-tRNA(Leu)
tRNA-Ile
L-Isoleucine 6.1.1.5 L-Isoleucyl-tRNA(Ile)
L-Lysine 6.1.1.6 L-Lysyl-tRNA(Lys)
Lysine biosynthesis
tRNA-Pyl
L-Pyrrolysine 6.1.1.26 L-Pyrrolysyl-tRNA(Pyl)
L-Arginine 6.1.1.19 L-Arginyl-tRNA(Arg)
Arginine and proline metabolism
L-Proline 6.1.1.15 L-Prolyl-tRNA(Pro)
Histidine metabolism
L-Histidine 6.1.1.21 L-Histidyl-tRNA(His)
L-Phenylalanine 6.1.1.20 L-Phenylalanyl-tRNA(Phe)
Phenylalanine, tyrosine and tryptophan biosynthesis
L-Tyrosine 6.1.1.1 L-Tyrosyl-tRNA(Tyr)
Tryptophan metabolism
L-Tryptophan 6.1.1.2 L-Tryptophanyl-tRNA(Trp)
00970 2/4/16
(c) Kanehisa Laboratories
